# Rapid and accurate sepsis diagnostics via a novel probe-based multiplex real-time PCR system

**DOI:** 10.1128/spectrum.00559-25

**Published:** 2025-09-30

**Authors:** Marco Favaro, Christian Fini, Maurizio Capannari, Ivano Petriccione, Claudia Rotondo, Assunta Navarra, Chiara Stellitano, Chiara De Giuli, Antonella Vulcano, Carla Fontana

**Affiliations:** 1 Dept Experimental Medicine, University of Rome Tor Vergata 175051, Rome, Italy; 2 Elettrobiochimica Srl, Rome, Italy; 3 National Institute for Infectious Diseases “L. Spallanzani” IRCCS, Rome, Italy; 4 Clinical Epidemiology Unit, National Institute for Infectious Diseases “L. Spallanzani” IRCCS, Rome, Italy; Inflammatix Inc., Sunnyvale, California, USA

**Keywords:** rapid diagnostics, pathogen identification, sepsis, antibiotic resistance genes

## Abstract

Sepsis is a critical clinical emergency that requires prompt diagnosis and intervention. Its prevalence has increased due to the aging population and increased antibiotic resistance. Early identification and the use of innovative technologies are crucial for improving patient outcomes. Modern methodologies are needed to minimize the turnaround time for diagnosis and improve outcomes. Rapid diagnostic tests and multiplex PCR are effective but have limitations in identifying a range of pathogens and target genes. Our study evaluated two novel probe-based multiplex real-time PCR systems: the SEPSI ID and SEPSI DR panels. These systems can quickly identify bacterial and fungal pathogens, alongside antibiotic resistance genes. The assays cover 29 microorganisms (gram-negative bacteria, gram-positive bacteria, yeast, and mold species), alongside 23 resistance genes and four virulence factors. A streamlined workflow uses 2 µL of broth from positive blood cultures (BCs) without nucleic acid extraction and provides results in approximately 1 h. We present the results from an evaluation of 228 BCs and 22 isolates previously characterized by whole-genome sequencing. In comparison to the reference methods, the SEPSI ID panel demonstrated a sensitivity of 96.88%, a specificity of 100%, and a PPV of 100%, whereas the SEPSI DR panel showed a sensitivity of 97.8%, a PPV of 89.7%, and a specificity of 96.7%. Both panels also identified additional pathogens and resistance-related targets not detected by conventional methods. This assay shows promise for rapidly and accurately diagnosing sepsis. Future studies should validate its performance in various clinical settings to enhance sepsis management and improve patient outcomes.

**IMPORTANCE:**

We present a new diagnostic method that enables the quick and precise identification of pathogens and resistance genes from positive blood cultures, eliminating the need for nucleic acid extraction. This technique can also be used on fresh pathogen cultures. It has the potential to greatly improve treatment protocols, leading to better patient outcomes, more responsible antibiotic use, and more efficient management of healthcare resources.

## INTRODUCTION

Sepsis and bloodstream infections (BSIs) are associated with significant morbidity and mortality rates (1). The early implementation of effective antimicrobial therapy, guided by rapid antimicrobial test results, has been shown to improve patient outcomes (1–3). Notably, in recent years, there has been a progressive increase in sepsis cases due to several factors, including aging, an increase in patients with complex clinical conditions and comorbidities, and the growing problem of antimicrobial resistance (AMR) (4). The COVID-19 pandemic has further exacerbated this situation (5). The impact of AMR on sepsis cannot be overstated (6–8). AMR, often termed the “silent pandemic” (9), frequently complicates the establishment of effective treatment, leading to increased morbidity and mortality, extended hospital stays, potential complications, and the occurrence of epidemic clusters. Additionally, AMR imposes substantial economic burdens due to the resulting need for more expensive drugs and procedures, prolonged hospitalization, and the potential to cause disability (10, 11). Addressing sepsis requires collaboration among clinicians, microbiologists, pharmacologists, and health directors to formulate an integrated strategy (12–14). Sepsis, as a time-dependent syndrome, represents a critical clinical and research emergency (15, 16). Consequently, laboratories must employ all available innovative technologies to enable rapid diagnosis (17, 18). In the diagnostic pathway for BSIs/sepsis, the appropriate and timely collection of blood cultures (BCs) is strongly recommended, as BC-based diagnostic methods remain the gold standard (19). Effective treatment of sepsis requires early recognition of clinical symptoms and access to a microbiology laboratory that uses advanced technologies for rapid testing. These rapid pathways provide timely diagnostic information, allowing clinicians to start targeted therapy quickly (18). Multiple studies have shown that technological advancements have focused primarily on rapid diagnostic tests (RDTs). However, syndromic panels and other multiplex PCR methods often fail to identify many pathogens and target resistance genes (20, 21). BC-free methods still lack sufficient sensitivity to be recommended as a replacement for BCs for detecting BSI pathogens (22). The ongoing implementation of next-generation sequencing (NGS) techniques, which are performed directly with blood or can be performed with BCs a few hours after incubation in a continuous monitoring incubation system, is promising (23). However, these techniques are currently the prerogative of a few large laboratories with access to advanced technologies that are generally expensive and require dedicated staff and great expertise, including bioinformatics (24). Therefore, at present, methods based on real-time PCR technologies still appear to be the simplest and most economically sustainable solution for most microbiological diagnostic laboratories. Hence, the development, validation, and evaluation of new RDTs are desirable.

The objective of our study was to evaluate a novel real-time PCR probe-based system for the rapid identification of bacterial and fungal pathogens, as well as the detection of target genes associated with resistance to major classes of antibiotics in patients with BSIs/sepsis.

## RESULTS

An overview of the SEPSI ID and SEPSI DR panel compositions, including the specific targets and fluorophores used in each multiplex reaction, is provided in Table 1 to support the results that follow.

**TABLE 1:** SEPSI DR and SEPSI ID panel composition^*a*,*b*,*c*,*d*,*e*,*f*,*g*^

Real-time PCR mixes included in the panels	Fluorophores				
	FAM	HEX	ROX	Cy5	Cy5.5
SEPSI DR					
Mix 1	*bla*_OXA48_	*bla*_VIM_	*bla*_KPC_	*bla*_NDM_	IC
Mix 2	*bla*_TEM_	*bla*_CTX-M_	*bla*_SHV_	*bla*_IMP_	IC
Mix 3	*bla*_OXA23_	*mcr*	*bla*_GES_	*bla*_CMY_	IC
Mix 4	*bla*_FIM_	AzolesRes	*van*A	*van*B	IC
Mix 5	*mec*C	OrfX	*mec*A	Panfungal	IC
Mix 6	*bla*_ampC_	*bla*_DHA-1_	*mgr*B	ompK36	IC
SEPSI ID					
Mix 1	*P. aeruginosa*	*K. pneumoniae*	*P. vulgaris/mirabilis*	*E. coli*	IC
Mix 2	*H. influenzae*	*K. oxytoca*	*E. cloacae*	*K. aerogenes*	IC
Mix 3	*L. pneumophila*	*S. marcescens*	*B. fragilis*	*A. baumannii*	IC
Mix 4	*S. malthophilia*	*E. faecalis*	*E. faecium*	*L. monocytogenes*	IC
Mix 5	*S. pyogenes*	*S. pneumoniae*	*S. agalactiae*	*N. meningitidis*	IC
Mix 6	*Staphylococcus* spp.	*A. fumigatus*	*S. aureus*	*C. freundii*	IC
Mix 7	*C. neoformans*	PVL	*C. albicans*	TSST	IC
Mix 8	*Candida* spp.	EXT a/b	*C. auris*	*K. pneumoniae* HMV	IC

^*a*^ AzoleRes detects the TR34/L98H azole resistance mutation in the cyp51A gene (25).

^*b*^ Panfungal includes pathogens from critical priority groups according to the WHO: *Cryptococcus neoformans*, *Aspergillus fumigatus*, *Candida albicans*, *Candida auris.*

^*c*^ *Candida* spp. includes *Nakaseomyces glabrata*, *Candida tropicalis,* and *Candida parapsilosis.*

^*d*^ PVL, panton valentine leukocidin; EXT a/b, exfoliative toxins a/b; TSST, toxic shock syndrome toxin.

^*e*^ HMV, *Hypermucoviscous K. pneumoniae*, specifically, the* rmp*A* and mag*A genes.

^*f*^ The list of identified *Staphylococcus* species is reported in File S1.

^*g*^ IC, internal control.

The SEPSI ID panel successfully identified a wide range of clinically significant pathogens commonly associated with sepsis. The most frequently detected bacteria were *Escherichia coli*, *Klebsiella pneumoniae*, *Pseudomonas aeruginosa*, *Staphylococcus aureus*, *Enterococcus faecium*, *Acinetobacter baumannii,* and *Proteus mirabilis*. The panel also detected the less common pathogens *Stenotrophomonas maltophilia*, *Enterococcus faecalis*, *Staphylococcus* spp. (the species are listed in File S1), and *Listeria monocytogenes*. These results were consistent with those of the reference methods (RFM), demonstrating the panel’s reliability in identifying these pathogens.

Specifically, the SEPSI ID panel was used to test 126 blood culture (BC) samples, including 100 positive and 26 negative cases. Four samples were excluded from the statistical analysis because the panel did not include their respective targets (namely, *Corynebacterium* spp., *Streptococcus gallolyticus*, and *Enterobacter roggenkampii*) (see File S1). Additionally, three samples were classified as false negatives: two *Enterobacter hormaechei* were not detected, and one *Enterococcus faecium* was missed in a mixed BC that tested positive for both *E. coli* and *E. faecium* by RFM.

Notably, the SEPSI ID panel identified two additional pathogens that were not detected by the RFM (Table 2).

**TABLE 2:** Identification of additional pathogens and resistance genes through the SEPSI ID/DR panels versus standard reference methods

Identification achieved through reference methods		Identification using the SEPSI ID/DR panel	Reference
Microbial identification			
*E. faecalis*		*E. faecalis, Staphylococcus* spp^*a*^.	See result no. 56 in File S1
*E. faecalis, E. hormaechei*		*E. faecalis, E. cloacae/hormaechei^b^, S. aureus*	See result no.58 in File S1
Mechanism of resistance detected			
*E. coli* ESBL^*c*^		*bla*_TEM_, *bla*_CTX-M_, *bla*_AmpC_	For AST^*g*^, see BC no. 18 in File S2
*E. faecalis* (susceptible to glycopeptides) + *K. pneumoniae* KPC		*bla*_KPC_, *bla*_SHV_, *bla*_CTX-M_	For AST^*g*^, see BCs no. 53 in File S2
*E. coli* KPC		*bla*_KPC_, *bla*_AmpC_	For AST^*g*^, see BC no. 56 in File S2
*E. coli* KPC		*bla*_KPC_, *bla*_AmpC_	For AST^*g*^, see BC no. 61 in File S2
*E. coli* overall susceptible		*bla*_AmpC_, *bla*_TEM_	For AST^*g*^, see BC no. 76 in File S2
*E. coli* ESBL^*c*^		*bla*_AmpC_, *bla*_TEM_	For AST^*g*^, see BC no. 74 in File S2
*S. aureus* MRSA^*d*^		*mec*A	For AST^*g*^, see BC no.8 in File S2
*E. coli* ESBL^*c*^		*bla*_CTX-M_, *bla*_AmpC_	For AST^*g*^, see BC no. 22 in File S2
*K. pneumoniae* KPC		*bla*_KPC_, *bla*_SHV_	For AST^*g*^, see BC no. 94 in File S2
*E. coli* ESBL^*c*^		*bla*_AmpC_, *bla*_TEM_	For AST^*g*^, see BC no. 63 in File S2
*K. pneumoniae* KPC		*bla*_KPC_, *bla*_TEM_, *bla*_SHV_, *bla*_CMY_	For AST^*g*^, see BC no. 19 in File S2
*K. pneumoniae* KPC		*bla*_KPC_, *bla*_SHV_, *bla*_TEM_	For AST^*g*^, see BC no. 26 in File S2
*K. pneumoniae* resistant to carbapenem^*e*^		*bla*_SHV_, *bla*_DHA^*f*^_	For AST^*g*^, see BC no. 14 in File S2
*K. pneumoniae* KPC		*bla*_KPC_, *bla*_SHV_, *bla*_TEM_, *bla*_GES_, *bla*_CMY_	For AST^*g*^, see BC no. 16 in File S2
*K. pneumoniae* KPC		*bla*_KPC_, *bla*_TEM_ *bla*_SHV_	For AST^*g*^, see BC no. 13 in File S2
*K. pneumoniae* KPC, NDM, OXA-48		*bla*_OXA48_, *bla*_NDM_, *bla*_KPC_, *bla*_SHV_, *bla*_CTX-M_, *bla*_TEM_, *bla*_CMY_	For AST^*g*^, see BC no. 44 in File S2
*K. pneumoniae* KPC		*bla*_KPC_, *bla*_SHV_, *bla*_CTX-M_, *bla*_TEM_	For AST^*g*^, see BC no. 9 in File S2

^*a*^ The list of *Staphylococcus* species, other than *S. aureus*, is reported in File S1.

^*b*^ The system identifies *E .cloacae* complex, including *E. hormachei, *but is unable to differentiate them.

^*c*^ ESBL: extended-spectrum beta-lactamases; the presence of ESBLs was established through automatic rules set in the Phoenix System (Becton Dickinson).

^*d*^ MRSA, methicillin-resistant *S. aureus.*

^*e*^ This BC was negative for NG-Test CARBA 5 (NG Biotech), which detects KPC, NMD, IMP, VIM, and OXA-48.

^*f*^ Resistance to carbapenem can also be inferred from the presence of *bla*_DHA _(26).

^*g*^ AST, antimicrobial susceptibility testing.

None of the 26 negative BCs produced a false positive (FP). Therefore, the system exhibited a sensitivity of 96.88% (95% CI: 95.58%–99.36%). The specificity and the PPV were 100% (95% CI: 86.77–100 and 96.09–100, respectively), indicating that all positive results were true positives. The negative predictive value (NPV) reached 89.66% (95% CI: 73.89–96.42), reflecting strong reliability in ruling out false negatives (FNs) Tables 3 and 4). The positive likelihood ratio (LHR+) was infinite due to the absence of false positives (i.e., 100% specificity), which makes the denominator of the formula equal to zero. This indicates an extremely high ability of the test to confirm the presence of the condition when the result is positive. The negative likelihood ratio (LHR−) was 0.03 (95% CI: 0.009–0.108), indicating that a negative test result is highly unlikely in a patient with this condition. The F1 score was 98.41%, reflecting high performance.

**TABLE 3:** Comparison of the SEPSI ID/DR panel results with the reference method outcomes

Result	SEPSI ID blood culture result			SEPSI DR blood culture resistance result		
	Positive	Negative	Total	Positive	Negative	Total
Positive	93	0	93	88	3	91
Negative	3	26	29	2	26	28
Total	96	26	122	90	29	119

**TABLE 4:** Diagnostic accuracy of SEPSI ID and SEPSI DR panels^*a*^

Measure of diagnostic accuracy	% (95% CI)	
	SEPSI ID blood culture	SEPSI DR blood culture resistance
Sensitivity	96.88 (95.58–99.36)	97.8 (92.2–99.7)
Specificity	100 (86.77–100)	89.7 (72.6–97.8)
PPV	100 (96.09–100)	96.7 (90.7–99.3)
NPV	89.66 (73.89–96.42)	92.9 (76.5–99.1)
LHR+	∞^*b*^	9.45 (3.24–27.6)
LHR-	0.03 (0.009–0.108)	0.02 (0.01–0.10)
Accuracy	97.54 (93.78–99.18)	95.8 (90.5–98.6)
F1 score	98.41	97.2

^*a*^ CI, confidence interval; PPV, positive predictive value; NPV, negative predictive value; LHR+, positive likelihood ratio; LHR−, negative likelihood ratio.

^*b*^ The infinite LHR⁺ reflects the absence of false positives (100% specificity), indicating the test’s exceptional ability to confirm the condition when positive.

The SEPSI DR panel was used to test a total of 128 BC samples, including 102 positive and 26 negative samples. The SEPSI DR panel identified a broader range of resistance mechanisms compared with the reference method, including several resistance genes such as *bla*_GES_ and *bla*_DHA_ that were not detected by conventional testing. Nine positive samples were excluded from the statistical evaluation. These samples were positive for yeasts whose target was not included among those detected by the “panfungal target.” Among the remaining 93 positive BCs tested using the SEPSI DR panel, 88 produced results that were consistent with those of the RFM, whereas five presented discrepancies. Three of the five discordant results were classified as FPs, and two were classified as FNs. Among the FPs, two samples tested positive for *E. coli* due to the presence of the *bla*_AmpC_ and *bla*_TEM_ genes. Still, they were phenotypically susceptible according to RFM, likely due to a lack of gene expression. This discrepancy may be explained by promoter mutations in *bla*_AmpC_, which can suppress gene expression, as reported in the literature (26–28). The third FP involved a *P. mirabilis* strain that carried the *bla*_CMY_ gene and appeared to be phenotypically unexpressed. FN was attributed to a culture that was positive for carbapenem-resistant *P. aeruginosa* but yielded only the *bla*_SHV_ gene. This is likely because carbapenem resistance in *P. aeruginosa* is often mediated by mechanisms such as porin loss/modification or efflux pump overexpression, pathways that are not targeted by the SEPSI DR panel nor by most commercial molecular assays. The last FN was a mixed infection with *E. coli* and vancomycin-resistant *E. faecium*. The SEPSI DR panel failed to detect the *van*A/B genes in this case. All the results are detailed in File S2. As expected, the 26 negative BCs yielded negative results.

The SEPSI DR panel had a sensitivity of 97.8% (95% CI: 92.2–99.7) and a specificity of 89.7% (95% CI: 72.6–97.8). The PPV was 96.7% (95% CI: 90.7–99.3), and the NPV was 92.9% (95% CI: 76.5–99.1) (Table 4). The LHR+ was 9.45, indicating that a positive result from the SEPSI DR panel provides strong evidence for the presence of resistance genes. The LHR− was 0.02 (95% CI: 0.01–0.10), indicating that a negative result from the SEPSI DR panel is highly reliable for ruling out the presence of resistance genes. This very low value suggests that the test is useful for confidently excluding antimicrobial resistance when no target is detected. The F1 score was 97.2%, reflecting a strong balance between PPV and sensitivity and confirming the panel’s overall diagnostic accuracy. Table 4 provides a comprehensive summary of the results obtained from both panels.

As shown in Table 1, the SEPSI DR panel revealed a wider range of resistance mechanisms, including genes such as *bla*_GES_ and *bla*_DHA_, that were not identified by the RFM (see also File S2). Importantly, the SEPSI DR panel primer design for TEM and SHV allows for the detection of both extended-spectrum β-lactamases (ESBLs) and narrow-spectrum β-lactamases, including *bla*_TEM-1/2_ and *bla*_SHV-1_. These enzymes are commonly found in *Enterobacterales* and confer resistance to penicillins and first-generation cephalosporins (28). This explains the resistance to ampicillin observed in two samples: one positive for *K. pneumoniae* harboring *bla*_SHV_ and the other positive for *P. mirabilis* harboring *bla*_TEM_ (see File S2). The AST results for reference strains are provided in File S2.

**Fig 1:**
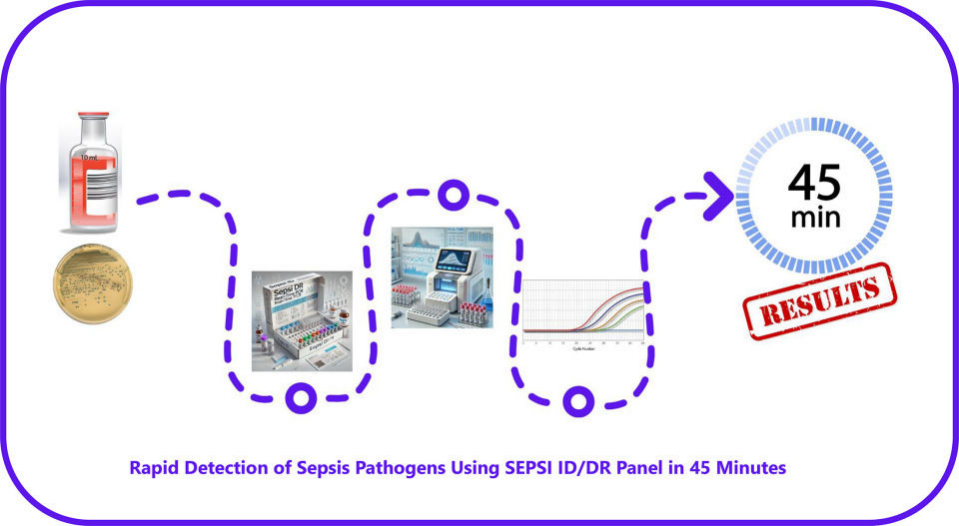
Rapid detection of sepsis pathogens using the SEPSI ID/DR panel.

Finally, Table 5 shows the SEPSI ID and SEPSI DR results for the ATCC reference strains and the isolates from our collection that were characterized using whole-genome sequencing (WGS) (all raw reads generated were submitted to the Sequence Read Archive (SRA) under the BioProjects ID PRJNA1243017 and PRJNA1259250). Although nearly all the expected targets were detected, WGS revealed additional resistance genes that were not covered by the SEPSI DR panel. Notably, the SEPSI DR assay successfully identified the *bla*_AmpC_ gene in *E. coli* ATCC 25922, which exhibited reduced susceptibility to cefoxitin, a phenotype also reported by Tracz et al. (27). Importantly, the results from both the SEPSI ID and SEPSI DR panels can be obtained in approximately 1 h, including approximately 45 min for the amplification step, with additional time required for data export and software-based analysis, resulting in a final turnaround time of approximately 1 h (see Fig. 1).

**TABLE 5:** Clinical isolate and ATCC characterization: resistance gene detection^*a*^^,^^*c*^

ID isolate^*b*^	Genes found through WGS	ID WGS sequence	Sepsi DR results
*E. faecium van*A	*van*HAX	TRCIO_01_S5_L001_R1_001.fastq.gz TRCIO_01_S5_L001_R2_001.fastq.gz	*van*A
*E. faecium van*A	*van*A, *van*X	TRCIO_16_S20_L001_R1_001.fastq.gz TRCIO_16_S20_L001_R2_001.fastq.gz	*van*A
*E. faecalis*	*van*B defective	TRCIO_17_S21_L001_R1_001.fastq.gz TRCIO_17_S21_L001_R2_001.fastq.gz	none
*E. faecium van*A	*van*HAX	TRCIO_18_S22_L001_R1_001.fastq.gz TRCIO_18_S22_L001_R2_001.fastq.gz	*van*A
*E. faecium van*A	*van*HAX	TRCIO_23_S27_L001_R1_001.fastq.gz TRCIO_23_S27_L001_R2_001.fastq.gz	*van*A
*E. faecium van*A	*van*HAX	1-Emo_S20_L001_R1_001.fastq.gz 1-Emo_S20_L001_R2_001.fastq.gz	*van*A
*E. faecium van*A	*van*HAX	2-Emo_S22_L001_R1_001.fastq.gz 2-Emo_S22_L001_R2_001.fastq.gz	*van*A
*E. faecium van*A	*van*HAX	ent-7emo_S19_L001_R1_001.fastq.gz ent-7emo_S19_L001_R2_001.fastq.gz	*van*A
*E. faecium van*A	*van*HAX	Entero-6emo_S7_L001_R1_001.fastq.gz Entero-6emo_S7_L001_R2_001.fastq.gz	*van*A
*E. faecium van*A	vanHAX	ent-3emo_S13_L001_R1_001.fastq.gz ent-3emo_S13_L001_R2_001.fastq.gz	*van*A
*E. faecium van*A	*van*HAX	Entero-4emo_S5_L001_R1_001.fastq.gz Entero-4emo_S5_L001_R2_001.fastq.gz	*van*A
*K. pneumoniae* NDM	*bla*_NDM-5_, *bla*_CTX-M-15_, *bla*_SHV-11_, *bla*_CMY-2_	NDM10_S10_L001_R1_001.fastq.gz NDM10_S10_L001_R2_001.fastq.gz	*bla*_NDM_, *bla*_TEM_, *bla*_CTX-M_, *bla*_SHV_, *bla*_CMY_
*K. pneumoniae* NDM	*bla*_NDM-1_, *bla*_CTX-M-15_, *bla*_OXA-1_, *bla*_OXA-9_, *bla*_TEM-1D_, *bla*_SHV-11_	NDM19_S19_L001_R1_001.fastq.gz NDM19_S19_L001_R2_001.fastq.gz	*bla*_NDM_, *bla*_TEM_, *bla*_CTX-M_, *bla*_SHV_
*K. pneumoniae* NDM	*bla*_NDM-1_, *bla*_CTX-M-15_, *bla*_OXA-1_, *bla*_OXA-9_, *bla*_TEM-1D_, *bla*_SHV-11_	NDM40_S1_L001_R1_001.fastq.gz NDM40_S1_L001_R2_001.fastq.gz	*bla*_NDM_, *bla*_TEM_, *bla*_CTX-M_, *bla*_SHV_
*K. pneumoniae* KPC	*bla*_KPC-3_, *bla*_SHV-100_	2023-KPC-Kpn-57_S1_L001_R1_001.fastq 2023-KPC-Kpn-57_S1_L001_R2_001.fastq	*bla*_KPC_, *bla*_SHV_
*A. baumannii*	*bla*_OXA-23_, *bla*_OXA-66_, *bla*_TEM-1D_, *bla*_ADC-25_	2023-Acibau-1_S17_L001_R1_001.fastq.gz 2023-Acibau-1_S17_L001_R2_001.fastq.gz	*bla*_TEM_, *bla*_OXA-23_
*A. baumannii*	*bla*_OXA-23_, *bla*_OXA-66_, *bla*_ADC-25_	2024-Acibau-3_S3_L001_R1_001.fastq.gz 2024-Acibau-3_S3_L001_R2_001.fastq.gz	*bla*_OXA-23_
*P. aeruginosa*	*bla*_IMP-13_, *bla*_OXA-50_, *bla*_PAO_	S20_L001_R1_001.fastq.gz S20_L001_R2_001.fastq.gz	*bla*_IMP_
*P. aeruginosa*	*bla*_VIM-2_, *bla*_OXA-486_, *bla*_PAO_	Pse-pro_S14_L001_R1_001.fastq.gz Pse-pro_S14_L001_R2_001.fastq.gz	*bla*_VIM_
*E. faecalis* VSE	na	na	none
*E. faecium* VSE	na	na	none
*S. aureus* MRSA	*mec*A	na	*mec*A, *orf*X
ATCC *K.pneumoniae* 700603	na	na	*bla*_SHV_
ATCC *K. pneumoniae* BAA-2814	na	na	*bla*_TEM_, *bla*_SHV_, *bla*_KPC_
ATCC *E. coli* 25922	na	na	*bla*_AmpC_
ATCC *E. faecalis* 29212	na	na	none
ATCC *S. aureus* 29213	na	na	*orf*X
ATCC *P. aeruginosa* 27853	na	na	none

^*a*^ VSE, vancomycin-susceptible enterococci.

^*b*^ Obtained using the standard-of-care testing.

^*c*^ na, WGS was not performed.

## DISCUSSION

AMR, often referred to as the “silent pandemic,” is a growing concern, making the effective treatment of sepsis more challenging (9). AMR leads to increased morbidity, mortality, and healthcare costs (29). Rapid diagnostic technologies, such as real-time PCR, are crucial for identifying pathogens quickly and accurately, allowing timely and targeted therapy (30). RDTs lead to better patient outcomes, optimize the use of medications, reduce selective pressure on microbial isolates, minimize the emergence of AMR, and shorten hospital stays (18, 31, 32).

This study aimed to evaluate the effectiveness of a new system based on real-time PCR probes for the rapid identification of bacterial and fungal pathogens, as well as the detection of antibiotic resistance genes, in BCs from patients with BSIs. Both the SEPSI ID and SEPSI DR panels demonstrated excellent diagnostic performance. Specifically, the SEPSI ID panel showed high sensitivity, specificity, and PPV. The NPV was 89.66%, and the F1 score reached 98.41%. This confirms the panel’s high accuracy in identifying both true positives and true negatives. Additionally, the low LHR value (0.03) indicates that a negative test result substantially reduces the likelihood of infection that can be detected by the SEPSI ID panel. This supports the test’s strong exclusion capability and indicates that a negative result is highly reliable for ruling out infection. Nevertheless, the SEPSI ID panel did not identify some pathogens, such as *Corynebacterium* spp., *S. gallolyticus,* and *E. roggenkampii*. Importantly, these pathogens, although clinically significant, are relatively less common. *Corynebacterium* spp. are increasingly recognized as opportunistic pathogens, particularly in immunocompromised patients. *S. gallolyticus* is associated with serious infections and an increased risk of colorectal cancer (33, 34). *E. roggenkampii* is a multidrug-resistant pathogen that poses a significant threat in healthcare settings (35). With a sensitivity of 97.8%, specificity of 89.7%, and PPV of 96.7%, the SEPSI DR panel demonstrated relevant diagnostic capability. Furthermore, an F1 score of 97.2% indicates a strong balance between PPV and sensitivity. Additionally, the high LHR+ value of 9.45 means that a positive result from the SEPSI DR panel strongly suggests the presence of resistance genes. This value approaches the threshold typically considered indicative of high diagnostic utility, thereby reinforcing the panel’s effectiveness in confirming antimicrobial resistance.

Importantly, the SEPSI DR panel identified several resistance genes not detected by conventional methods, including *bla*_GES_ and *bla*_DHA_, underscoring its broader detection capability.

Expanding diagnostic coverage improves the overall treatment approach by enabling earlier and more precise therapeutic decisions. Timely and targeted antimicrobial therapy has been shown to accelerate infection resolution, leading to shorter hospital stays and improved patient outcomes (36, 37). Furthermore, encouraging the appropriate use of antimicrobials helps reduce the selective pressure that drives the emergence of AMR (38). In this context, the SEPSI DR panel has the potential to contribute to antimicrobial stewardship by providing detailed resistance profiles that could support more informed antibiotic selection and potentially help preserve the long-term efficacy of antibiotics. It is possible that the panel might also help mitigate the development and spread of AMR by reducing reliance on broad-spectrum agents. Although our study did not directly compare the reporting times of the standard of care and the new RDT, notably, the novel system delivers results in approximately 1 h. In contrast, traditional AST typically requires 24−48 h (39), depending on the method used and the microorganism involved. This substantial reduction in turnaround time has the potential to significantly accelerate clinical decision-making in the management of sepsis. This is crucial in sepsis management, where every hour of delay in appropriate treatment increases mortality risk (36, 37).

Despite these promising results, this study has several limitations. First, the sample size was relatively small, which may limit the generalizability of the findings. Larger studies are needed to confirm these results and provide more robust data. Second, the study was conducted in a single laboratory setting; hence, the results may not reflect the performance of the diagnostic panels in different clinical environments with varying levels of expertise and resources. Third, the SEPSI ID panel did not identify certain pathogens, indicating that the panel coverage is not comprehensive. It would be beneficial to include these pathogens in the SEPSI ID panel to ensure comprehensive identification and effective treatment and management of infections. Additionally, the SEPSI DR panel exhibited lower specificity due to some FP results. This is a limitation of all molecular systems that can potentially detect resistance genes that may not be expressed, which can lead to unnecessary antibiotic treatments. To address this issue, it is important to share knowledge of the limitations of molecular systems with clinicians. Finally, the study did not evaluate the cost-effectiveness of implementing these diagnostic panels in routine clinical practice, which is an important consideration for widespread adoption. Future research should address these limitations by including larger, multicenter studies; expanding the range of detectable pathogens and resistance genes; and assessing the cost-effectiveness of these diagnostic tools in various healthcare settings.

In conclusion, despite some limitations, SEPSI ID/DR panels are highly effective at rapidly and accurately identifying pathogens and their resistance target genes, making them an invaluable asset in modern diagnostics. Their rapid performance accelerates decision-making, playing a pivotal role in improving patient care and outcomes. Adopting this technology can greatly increase clinical effectiveness and ensure timely treatment.

## MATERIALS AND METHODS

### SEPSI ID and SEPSI DR description

The system being evaluated is currently undergoing CE/IVDR certification and is intended for research use only. It comprises a qualitative, multiplex real-time PCR probe-based assay consisting of two diagnostic kits: SEPSI ID and SEPSI DR. These kits were developed in collaboration with the Clinical Microbiology Laboratory at the University of Rome “Tor Vergata” and Elettrobiochimica Srl. SEPSI ID and SEPSI DR are single-test systems that include a mixture of buffer, Taq DNA polymerase, primers, and probes in a prealiquoted liquid phase. The tests are divided into two strips, with six tubes for the DR test and eight tubes for the ID test, which are necessary for multiplex real-time PCR (Table 1). The strips included all the required liquid reagents and were stored at −20°C until use. Before testing, the reagents were thawed, and 2 µL of positive BC was added (nucleic acid extraction was not necessary). The primers and probes were sourced from Eurofins (Eurofins Genomics Europe; Ebensburg, Germany). In each tube, up to five targets (four targets plus an internal control) can be detected simultaneously. Five probes labeled with different fluorophores were used. The fluorophores used were FAM, ROX, Cy5, HEX, and Cy5-5, which were attached to the 5' end of the probe, whereas a nonfluorescent quencher, BHQ, was located at the 3' end. The system ensures the absence of amplification reaction inhibitors through the use of an internal control that amplifies and detects a human gene, specifically β-actin.

Specifically, the SEPSI ID/DR panel can be used to identify a total of 29 microorganisms, including 15 gram-negative and eight gram-positive bacteria, six species of yeast and mold (including a panfungal target), 23 resistance genes, and four virulence genes. Amplifications were conducted using the Bio-Rad CFX96 system (Bio-Rad, Hercules, California, USA). The assay was also validated on the CFX Opus 96 Real-Time System and the Bio-Rad CFX96 Touch system (Bio-Rad). The Taq polymerase used was Air Dryable 4X Direct DNA qPCR Blood (Meridian Bioscience, Cincinnati, Ohio, USA). Both panels provided the final results within 1 h.

The primer and probe sequences are not disclosed due to intellectual property rights protection by the University of Study of Rome “Tor Vergata” under Patent No. 102020000018400 and Patent No. 10200000018400.

The selection of target organisms and AMR/virulence genes for the panels was based on their clinical relevance in BSIs and their association with AMR. The panel includes ESKAPE pathogens and other key bacteria, which were selected on the basis of global and Italian epidemiological data (39, 40). Resistance gene targets were chosen to cover the most impactful mechanisms encountered in clinical settings. These genes include the following β-lactamase genes: *bla*_KPC_, *bla*_NDM_, *bla*_VIM_, *bla*_IMP_, *bla*_OXA-48_, *bla*_OXA-23_, *bla*_TEM_, *bla*_SHV_, *bla*_CTX-M_, *bla*_GES_, *bla*_CMY_, *bla*_AmpC_, and *bla*_DHA-1_. These genes are associated with resistance to carbapenems and extended-spectrum cephalosporins. Additional targets include *mcr* for colistin resistance; *van*A and *van*B for vancomycin resistance; and *mec*A, *mec*C, and *Orf*X for methicillin resistance (41). The *mgr*B gene was included because of its role in colistin resistance, and the *omp*K36 gene was included because of its contribution to membrane impermeability, particularly in *K. pneumoniae*. The panel also includes markers for azole resistance and a panfungal target to broaden diagnostic coverage. Virulence genes (Panton-Valentine leukocidin, exfoliative toxins A/B, toxic shock syndrome toxin, *rmp*A and *mag*A) were selected for their association with increased pathogenicity and adverse clinical outcomes (42, 43). This ensures that the panel supports the rapid provision of actionable diagnostic information for managing sepsis. The reproducibility study for both panels is detailed in File S3. The results of cross-reactivity tests performed to detect targets individually or in combination (polymicrobial) are reported in File S4.

### Samples tested

This study included patients admitted to our hospital with suspected sepsis or BSIs. All positive BC samples from adult patients aged 18 years and older were analyzed prospectively. These samples were processed in batches daily and stored at 4°C until the results were obtained.

The performance of the system was evaluated by including a total of 202 positive BCs: 100 were tested with the SEPSI ID kit, and 102 were tested with the SEPSI DR. To rule out the possibility of false-positive results, we tested 26 negative BCs using both panels. We assessed the performance of the new system alongside RFM. To assess the ability of the SEPSI DR panel to detect resistance genes, this study included 22 isolates from our collection and six ATCC strains. Of these 22 isolates, 19 had been previously characterized through WGS analysis (data not shown), whereas the remaining three had not been sequenced.

The ATCC strains included were *E. faecalis* ATCC 29212, *K. pneumoniae* ATCC 700603, *K. pneumoniae* BAA-2814, *S. aureus* ATCC 28213, *E. coli* ATCC 25922, and *Pseudomonas aeruginosa* ATCC 27853. The antimicrobial susceptibility profiles of these strains are well documented.

### PCR assay conditions: protocol for processing positive BC broth

Broth from positive BC (2 µL) was added directly to each well of the strip, and a multichannel pipette was used to streamline the process. The strips were then sealed with the appropriate optical caps. Amplification was conducted using a CFX96 system (Bio-Rad) according to the established protocol: one initial step at 95°C for 3 min, followed by 35 cycles of 95°C for 10 s and 60°C for 25 s. After amplification, the results were exported using the “Export LIMS Folder” function, and data analysis was performed using EBC software (for Bio-Rad instruments only). The EBC software is designed to analyze real-time PCR data generated by the SEPSI ID and SEPSI DR panels, which are used either individually or in combination. Following the amplification step, the operator exports the data for processing by the software, which then generates a comprehensive report including both pathogen identification and interpretation of the associated resistance profile. Currently, the protocol has only been validated on the CFX96 system (Bio-Rad).

### PCR assay conditions: protocol for processing bacterial isolates

Although the test was optimized for use with a positive BC sample, it can also be applied to fresh colonies of bacterial isolates. This can be accomplished by preparing a bacterial suspension in sterile distilled water corresponding to 0.5 on the McFarland scale. The PCR assay was conducted with 2 µL of the prepared bacterial suspension following the aforementioned BC protocols.

### Reference methods

BC-based methods remain the gold standard for the isolation and detection of pathogens involved in BSIs (19). BCs were obtained from patients in whom a physician suspected infection (BSI/sepsis), and for adult patients, two sets of BCs (with each set composed of aerobic and anaerobic bottles) were routinely inoculated at the bedside and immediately delivered to our microbiology laboratory. Upon arrival at the laboratory, the BCs were incubated in a BACTEC FX system (Becton Dickinson; Sparks, MD, USA), and those identified as positive were subcultured and Gram-stained (smear microscopy). Specifically, aliquots (10 µL) of broth from the positive bottles were plated onto chocolate agar, blood agar, Columbia CNA agar, Sabouraud dextrose agar, and MacConkey agar (Thermo Fisher Scientific; Waltham, MA, USA) using the WASP automated system (Copan, Brescia, Italy). The plates were incubated under aerobic and anaerobic conditions at 37°C for 24 h. The growth of the bacterial colonies was monitored, and the bacterial species were identified using the MALDI-TOF assay (MALDI TOF Syrius; Bruker Daltonics, Bremen, Germany). Antimicrobial susceptibility testing (AST) was performed using the Phoenix system (Becton Dickinson). The AST results were interpreted according to EUCAST clinical breakpoints v14.0 (EUCAST available at https://www.eucast.org/clinical_breakpoints). The identification of carbapenemase-producing *Enterobacterales* (CPE) and *P. aeruginosa* was performed using immunochromatographic assays (NG-Test CARBA 5 (NG Biotech, Guipry, France). For some cases, particularly critically ill patients, upon detection of positivity, the BC was also processed using BioFire FilmArray BCID2 (bioMérieux, Las Balmas, France) (File S2), which allows the identification of pathogens as well as major resistance-related targets (44).

### Statistical evaluation

Statistical analyses were conducted to evaluate the performance of the diagnostic test. Specifically, we computed sensitivity, specificity, PPV, NPV, LHR+, and LHR− to assess the probability of test results based on the presence or absence of the condition. Overall, the PPV was calculated as the proportion of correctly identified cases among the total number tested. The F1 score, which indicates a balance between PPV and sensitivity, was calculated using standard statistical methods (45).

## SUPPLEMENTAL MATERIAL

Data S1 spectrum.00559-25-s0001.xlsx

Data S2 spectrum.00559-25-s0002.xlsx

Data S3 spectrum.00559-25-s0003.docx

Data S4 spectrum.00559-25-s0004.xlsx

ASM does not own the copyrights to Supplemental Material that may be linked to, or accessed through, an article. The authors have granted ASM a non-exclusive, world-wide license to publish the Supplemental Material files. Please contact the corresponding author directly for reuse.

## References

[1] La Via L, Sangiorgio G, Stefani S, Marino A, Nunnari G, Cocuzza S, La Mantia I, Cacopardo B, Stracquadanio S, Spampinato S, Lavalle S, Maniaci A. 2024. The global burden of sepsis and septic shock. Epidemiologia (Basel) 5:456–478. doi:10.3390/epidemiologia5030032 39189251 PMC11348270

[2] Rudd KE, Kissoon N, Limmathurotsakul D, Bory S, Mutahunga B, Seymour CW, Angus DC, West TE. 2018. The global burden of sepsis: barriers and potential solutions. Crit Care 22:232. doi:10.1186/s13054-018-2157-z 30243300 PMC6151187

[3] Rhee C, Dantes R, Epstein L, Murphy DJ, Seymour CW, Iwashyna TJ, Kadri SS, Angus DC, Danner RL, Fiore AE, Jernigan JA, Martin GS, Septimus E, Warren DK, Karcz A, Chan C, Menchaca JT, Wang R, Gruber S, Klompas M, CDC Prevention Epicenter Program. 2017. Incidence and trends of sepsis in US hospitals using clinical vs claims data, 2009-2014. JAMA 318:1241–1249. doi:10.1001/jama.2017.13836 28903154 PMC5710396

[4] Shafaati M, Salehi M, Zare M. 2024. The twin challenges of longevity and climate change in controlling antimicrobial resistance. J Antibiot (Tokyo) 77:399–402. doi:10.1038/s41429-024-00730-6 38724628

[5] Shappell C, Rhee C, Klompas M. 2023. Update on sepsis epidemiology in the era of COVID-19. Semin Respir Crit Care Med 44:173–184. doi:10.1055/s-0042-1759880 36646093

[6] Rudd KE, Johnson SC, Agesa KM, Shackelford KA, Tsoi D, Kievlan DR, Colombara DV, Ikuta KS, Kissoon N, Finfer S, Fleischmann-Struzek C, Machado FR, Reinhart KK, Rowan K, Seymour CW, Watson RS, West TE, Marinho F, Hay SI, Lozano R, Lopez AD, Angus DC, Murray CJL, Naghavi M. 2020. Global, regional, and national sepsis incidence and mortality, 1990-2017: analysis for the global burden of disease study. Lancet 395:200–211. doi:10.1016/S0140-6736(19)32989-7 31954465 PMC6970225

[7] Macias J, Kahly O, Pattik-Edward R, Khan S, Qureshi A, Shaik A, Shala A, Shah D. 2022. Sepsis: a systematic review of antibiotic resistance and antimicrobial therapies. MRI 11:9–23. doi:10.4236/mri.2022.112002

[8] Debela N, Nekahiwot S. 2024. Sepsis, antimicrobial resistance, and alternative therapies. Am J Health Res 12:8–18. doi:10.11648/j.ajhr.20241201.12

[9] Nature Communications. 2024. Antimicrobial resistance: a silent pandemic. Nat Commun 15:6198. doi:10.1038/s41467-024-50457-z 39043632 PMC11266683

[10] Ahmed SK, Hussein S, Qurbani K, Ibrahim RH, Fareeq A, Mahmood KA, Mohamed MG. 2024. Antimicrobial resistance: impacts, challenges, and future prospects. J Med Surg Pub Health 2:100081. doi:10.1016/j.glmedi.2024.100081

[11] Naghavi M, Vollset SE, Ikuta KS, Swetschinski LR, Gray AP, Wool EE, Robles Aguilar G, Mestrovic T, Smith G, Han C, et al.. 2024. Global burden of bacterial antimicrobial resistance 1990–2021: a systematic analysis with forecasts to 2050. Lancet 404:1199–1226. doi:10.1016/S0140-6736(24)01867-1 39299261 PMC11718157

[12] Tedesco ER, Whiteman K, Heuston M, Swanson-Biearman B, Stephens K. 2017. Interprofessional collaboration to improve sepsis care and survival within a tertiary care emergency department. J Emerg Nurs 43:532–538. doi:10.1016/j.jen.2017.04.014 28550958

[13] Halstead DC. 2022. Can multidisciplinary sepsis teams help solve a global concern? a review of the literature. Clin Microbiol Newsl 44:131–137. doi:10.1016/j.clinmicnews.2022.08.001

[14] Pan American Health Organization. 2024. CE174.R2 - strategy and plan of action to decrease the burden of sepsis through an integrated approach 2025–2029. https://www.paho.org/en/documents/ce174r2-strategy-and-plan-action-decrease-burden-sepsis-through-integrated-approach-2025.

[15] Miller JM, Binnicker MJ, Campbell S, Carroll KC, Chapin KC, Gonzalez MD, Harrington A, Jerris RC, Kehl SC, Leal SM Jr, Patel R, Pritt BS, Richter SS, Robinson-Dunn B, Snyder JW, Telford S III, Theel ES, Thomson RB Jr, Weinstein MP, Yao JD. 2024. Guide to utilization of the microbiology laboratory for diagnosis of infectious diseases: 2024 update by the infectious diseases society of America (IDSA) and the American society for microbiology (ASM). Clin Infect Dis 5:ciae104. doi:10.1093/cid/ciae104 38442248

[16] National Institute for Health and Care Excellence. 2016. Suspected sepsis: recognition, diagnosis and early management. NICE Guideline, NG51. 32011837

[17] Eubank TA, Long SW, Perez KK. 2020. Role of rapid diagnostics in diagnosis and management of patients with sepsis. J Infect Dis 222:S103–S109. doi:10.1093/infdis/jiaa263 32691836

[18] Tiseo G, Brigante G, Giacobbe DR, Maraolo AE, Gona F, Falcone M, Giannella M, Grossi P, Pea F, Rossolini GM, Sanguinetti M, Sarti M, Scarparo C, Tumbarello M, Venditti M, Viale P, Bassetti M, Luzzaro F, Menichetti F, Stefani S, Tinelli M, The Italian Society of Anti-Infective Therapy (SITA), the Italian Group for Antimicrobial Stewardship (GISA), the Italian Association of Clinical Microbiologists (AMCLI) and the Italian Society of Microbiology (SIM). Int J Antimicrob Agents. 2022. Diagnosis and management of infections caused by multidrug-resistant bacteria: guideline endorsed by the Italian Society of Infection and Tropical Diseases (SIMIT). Int J Antimicrob Agents 60:106611. doi:10.1016/j.ijantimicag.2022 35697179

[19] Lamy B, Sundqvist M, Idelevich EA. 2020. ESCMID study group for bloodstream infections, endocarditis and sepsis (ESGBIES). Clin Microbiol Infect 26:142–150. doi:10.1016/j.cmi.2019.11.017 31760113

[20] Dumkow LE, Worden LJ, Rao SN. 2021. Syndromic diagnostic testing: a new way to approach patient care in the treatment of infectious diseases. J Antimicrob Chemother 76:iii4–iii11. doi:10.1093/jac/dkab245 34555157 PMC8460095

[21] Ryu H, Abdul Azim A, Bhatt PJ, Uprety P, Mohayya S, Dixit D, Kirn TJ, Narayanan N. 2023. Rapid diagnostics to enhance therapy selection for the treatment of bacterial infections. Curr Pharmacol Rep 9:198–216. doi:10.1007/s40495-023-00323-1 40161380 PMC11951845

[22] Rapszky GA, Do To UN, Kiss VE, Kói T, Walter A, Gergő D, Meznerics FA, Rakovics M, Váncsa S, Kemény LV, Csupor D, Hegyi P, Filbin MR, Varga C, Fenyves BG. 2025. Rapid molecular assays versus blood culture for bloodstream infections: a systematic review and meta-analysis. EClinicalMedicine 79:103028. doi:10.1016/j.eclinm.2024.103028 39968206 PMC11833021

[23] Ali J, Johansen W, Ahmad R. 2024 Short turnaround time of seven to nine hours from sample collection until informed decision for sepsis treatment using nanopore sequencing. Sci Rep 14:6534. doi:10.1038/s41598-024-55635-z 38503770 PMC10951244

[24] Lee IK, Chang JP, Huang WC, Tai CH, Wu HT, Chi CH. 2022. Comparative of clinical performance between next-generation sequencing and standard blood culture diagnostic method in patients suffering from sepsis. J Microbiol Immunol Infect 55:845–852. doi:10.1016/j.jmii.2022.07.011 35995673

[25] Berkow EL, Nunnally NS, Bandea A, Kuykendall R, Beer K, Lockhart SR. 2018. Detection of TR_34_/L98H CYP51A mutation through passive surveillance for azole-resistant Aspergillus fumigatus in the United States from 2015 to 2017. Antimicrob Agents Chemother 62:e02240-17. doi:10.1128/AAC.02240-17 29463545 PMC5923109

[26] Chudácková E, Bergerová T, Fajfrlík K, Cervená D, Urbásková P, Empel J, Gniadkowski M, Hrabák J. 2010. Carbapenem-nonsusceptible strains of Klebsiella pneumoniae producing SHV-5 and/or DHA-1 beta-lactamases in a Czech hospital. FEMS Microbiol Lett 309:62–70. doi:10.1111/j.1574-6968.2010.02016.x 20528936

[27] Tracz DM, Boyd DA, Bryden L, Hizon R, Giercke S, Van Caeseele P, Mulvey MR. 2005. Increase in ampC promoter strength due to mutations and deletion of the attenuator in a clinical isolate of cefoxitin-resistant Escherichia coli as determined by RT-PCR. J Antimicrob Chemother 55:768–772. doi:10.1093/jac/dki074 15761065

[28] Tooke CL, Hinchliffe P, Bragginton EC, Colenso CK, Hirvonen VHA, Takebayashi Y, Spencer J. 2019. β-lactamases and β-lactamase inhibitors in the 21st century. J Mol Biol 431:3472–3500. doi:10.1016/j.jmb.2019.04.002 30959050 PMC6723624

[29] Huemer M, Mairpady Shambat S, Brugger SD, Zinkernagel AS. 2020. Antibiotic resistance and persistence-Implications for human health and treatment perspectives. EMBO Rep 21:e51034. doi:10.15252/embr.202051034 33400359 PMC7726816

[30] Peri AM, Stewart A, Hume A, Irwin A, Harris PNA. 2021. New microbiological techniques for the diagnosis of bacterial infections and sepsis in ICU including point of care. Curr Infect Dis Rep 23:12. doi:10.1007/s11908-021-00755-0 34149321 PMC8207499

[31] Timbrook TT, Morton JB, McConeghy KW, Caffrey AR, Mylonakis E, LaPlante KL. 2017. The effect of molecular rapid diagnostic testing on clinical outcomes in bloodstream infections: a systematic review and meta-analysis. Clin Infect Dis 64:15–23. doi:10.1093/cid/ciw649 27678085

[32] Mencacci A, De Socio GV, Pirelli E, Bondi P, Cenci E. 2023. Laboratory automation, informatics, and artificial intelligence: current and future perspectives in clinical microbiology. Front Cell Infect Microbiol 13:1188684. doi:10.3389/fcimb.2023.1188684 37441239 PMC10333692

[33] Yamamuro R, Hosokawa N, Otsuka Y, Osawa R. 2021. Clinical characteristics of Corynebacterium bacteremia caused by different species, Japan, 2014-2020. Emerg Infect Dis 27:2981–2987. doi:10.3201/eid2712.210473 34812137 PMC8632174

[34] Abdulamir AS, Hafidh RR, Abu Bakar F. 2011. The association of Streptococcus bovis/gallolyticus with colorectal tumors: the nature and the underlying mechanisms of its etiological role. J Exp Clin Cancer Res 30:11. doi:10.1186/1756-9966-30-11 21247505 PMC3032743

[35] Tenover FC, Tickler IA. 2024. Genomic analysis of Enterobacter species isolated from patients in United States hospitals. Antibiotics (Basel) 13:865. doi:10.3390/antibiotics13090865 39335038 PMC11428811

[36] Liu VX, Fielding-Singh V, Greene JD, Baker JM, Iwashyna TJ, Bhattacharya J, Escobar GJ. 2017. The timing of early antibiotics and hospital mortality in sepsis. Am J Respir Crit Care Med 196:856–863. doi:10.3389/fcimb.2023.1188684 28345952 PMC5649973

[37] Kumar NR, Balraj TA, Kempegowda SN, Prashant A. 2024. Multidrug-resistant sepsis: a critical healthcare challenge. Antibiotics (Basel) 13:46. doi:10.3390/antibiotics13010046 38247605 PMC10812490

[38] Nguyen-Thanh L, Wernli D, Målqvist M, Graells T, Jørgensen PS. 2024. Characterising proximal and distal drivers of antimicrobial resistance: an umbrella review. J Glob Antimicrob Resist 36:50–58. doi:10.1016/j.jgar.2023.12.008 38128730

[39] Edmondson R, Saeed K, Green S, O’Dwyer M. 2024. Improving turnaround times for routine antimicrobial sensitivity testing following European committee on antimicrobial susceptibility testing methodology in patients with bacteraemia. Antibiotics (Basel) 13:1094. doi:10.3390/antibiotics13111094 39596787 PMC11591232

[40] Foglia F, Della Rocca MT, Melardo C, Nastri BM, Manfredini M, Montella F, De Filippis A, Finamore E, Galdiero M. 2023. Bloodstream infections and antibiotic resistance patterns: a six-year surveillance study from southern Italy. Pathog Glob Health 117:381–391. doi:10.1080/20477724.2022.2129161 36190133 PMC10177691

[41] Banerjee R, Patel R. 2023. Molecular diagnostics for genotypic detection of antibiotic resistance: current landscape and future directions. JAC Antimicrob Resist 5:dlad018. doi:10.1093/jacamr/dlad018 36816746 PMC9937039

[42] Pan Y, Zeng J, Li L, Yang J, Tang Z, Xiong W, Li Y, Chen S, Zeng Z. 2020. Coexistence of antibiotic resistance genes and virulence factors deciphered by large-scale complete genome analysis. mSystems 5:e00821-19. doi:10.1128/msystems.00821-19 PMC8534731 32487745

[43] Karmakar A, Jana D, Dutta K, Dua P, Ghosh C. 2018. Prevalence of panton-valentine leukocidin gene among community acquired Staphylococcus aureus: a real-time PCR study. J Pathog 2018:4518541. doi:10.1155/2018/4518541 30245888 PMC6139182

[44] Berinson B, Both A, Berneking L, Christner M, Lütgehetmann M, Aepfelbacher M, Rohde H. 2021. Usefulness of bioFire filmArray BCID2 for blood culture processing in clinical practice. J Clin Microbiol 59:e0054321. doi:10.1128/JCM.00543-21 33980648 PMC8373244

[45] Altman DG. 1991. Practical statistics for medical research. Chapman and Hall, London.

