# Supplementary material for: Rapid and accurate sepsis diagnostics via a novel probe-based multiplex real-time PCR system
Source: Microbiol Spectr. 2025 Sep 30;13(11):e00559-25. doi: 10.1128/spectrum.00559-25 (PMC12584680; doi:10.1128/spectrum.00559-25)
Supplement: Data S3 [file spectrum.00559-25-s0003.docx]

**Supplementary File 3.** **The reproducibility study for both panels.**

Reproducibility was evaluated by determining the concentration of 1000 molecules for each target (LOD). Plasmids with a known concentration were treated under the same conditions as the diagnostic test and used as a reference.

Values from five repetitions of the test are reported.

The data are summarised in Tables A and B for DR and ID, respectively.

Table A

| **Target** | **Mix** | **CT in average % Pos. Replicates (Total)** | **The percentage of replicates that were successful out of a total of five.** |
| --- | --- | --- | --- |
|  | **Mix 1** |  |  |
| OXA-48 |  | 26.31 | 100% (5/5) |
| KPC |  | 25.49 | 100% (5/5) |
| NDM |  | 21.33 | 100% (5/5) |
| VIM |  | 26.93 | 100% (5/5) |
| CI |  | 26.18 | 100% (5/5) |
|  | **Mix 2** |  |  |
| TEM |  | 25.07 | 100% (5/5) |
| SHV |  | 26.00 | 100% (5/5) |
| IMP |  | 27.64 | 100% (5/5) |
| CTX-M |  | 26.18 | 100% (5/5) |
| CI |  | 27.64 | 100% (5/5) |
|  | **Mix 3** |  |  |
| OXA-23 |  | 24.56 | 100% (5/5) |
| GES |  | 27.46 | 100% (5/5) |
| CMY |  | 27.54 | 100% (5/5) |
| MCR (COLISTINA) |  | 26.08 | 100% (5/5) |
| CI |  | 28.66 | 100% (5/5) |
|  | **Mix 4** |  |  |
| FIM |  | 27.68 | 100% (5/5) |
| VAN A |  | 27.79 | 100% (5/5) |
| VAN B |  | 27.41 | 100% (5/5) |
| *Aspergillus* resistance |  | 31.89 | 100% (5/5) |
| CI |  | 28.35 | 100% (5/5) |
|  | **Mix 5** |  |  |
| ORF X |  | 28.67 | 100% (5/5) |
| MEC C |  | 29.42 | 100% (5/5) |
| MEC A |  | 26.62 | 100% (5/5) |
| *C. albicans* |  | 27.22 | 100% (5/5) |
| CI |  | 28.47 | 100% (5/5) |
|  | **Mix 6** |  |  |
| DHA_1 |  | 29.40 | 100% (5/5) |
| OMP36 |  | 29.52 | 100% (5/5) |
| AMP-C |  | 28.42 | 100% (5/5) |
| MGR-B |  | 26.49 | 100% (5/5) |
| CI |  | 28.50 | 100% (5/5) |

Table B

| **Target** | **Mix** | **CT in average % Pos. Replicates (Total)** | **The percentage of replicates that were successful out of a total of five.** |
| --- | --- | --- | --- |
|  | **Mix 1** |  |  |
| *P. aeruginosa* |  | 26.25 | 100% (5/5) |
| *P. mirabilis* |  | 24.50 | 100% (5/5) |
| *P. vulgaris* |  | 24.40 | 100% (5/5) |
| *E. coli* |  | 34.14 | 100% (5/5) |
| CI |  | 28.42 | 100% (5/5) |
|  | **Mix 2** |  |  |
| *H. influenzae* |  | 32.07 | 100% (5/5) |
| *K. oxytoca* |  | 26.80 | 100% (5/5) |
| *E. cloacae* |  | 30.16 | 100% (5/5) |
| *K. aerogenes* |  | 27.39 | 100% (5/5) |
| CI |  | 28.88 | 100% (5/5) |
|  | **Mix 3** |  |  |
| *L. pneumophila* |  | 32.38 | 100% (5/5) |
| *S. marcescens* |  | 29.20 | 100% (5/5) |
| *B. fragilis* |  | 31.38 | 100% (5/5) |
| *A. baumannii* |  | 27.98 | 100% (5/5) |
| CI |  | 28.66 | 100% (5/5) |
|  | **Mix 4** |  |  |
| *S. maltophilia* |  | 31.80 | 100% (5/5) |
| *E. faecalis* |  | 29.50 | 100% (5/5) |
| *E. faecium* |  | 31.01 | 100% (5/5) |
| *L. monocytogenes* |  | 31.89 | 100% (5/5) |
| CI |  | 28.35 | 100% (5/5) |
|  | **Mix 5** |  |  |
| *S. pyogenes* |  | 25.52 | 100% (5/5) |
| *S. pneumoniae* |  | 25.81 | 100% (5/5) |
| *S. agalactiae* |  | 31.43 | 100% (5/5) |
| *N. meningitidis* |  | 25.69 | 100% (5/5) |
| CI |  | 28.47 | 100% (5/5) |
|  | **Mix 6** |  |  |
| *Staphylococcus* spp. |  | 26.47 | 100% (5/5) |
| *S. aureus* |  | 29.16 | 100% (5/5) |
| *A. fumigatus* |  | 24.15 | 100% (5/5) |
| *Citrobacter freundii* |  | 26.21 | 100% (5/5) |
| CI |  | 28.98 | 100% (5/5) |
|  | **Mix 7** |  |  |
| *C. neoformans* |  | 30.65 | 100% (5/5) |
| *S. aureus* PVL |  | 33.50 | 100% (5/5) |
| *S. aureus* TSST |  | 31.58 | 100% (5/5) |
| *C. albicans* |  | 30.76 | 100% (5/5) |
| CI |  | 28.40 | 100% (5/5) |
|  | **Mix 8** |  |  |
| *C. parapsilosis* |  | 25.69 | 100% (5/5) |
| *C. tropicalis* |  | 28.40 | 100% (5/5) |
| *C. glabrata (Nakasomyces glabrata)* |  | 28.34 | 100% (5/5) |
| *S. aureus* exfoliative toxins a/b |  | 27.73 | 100% (5/5) |
| *K. pneumoniae* (hypermucoviscous) *rmp*A |  | 28.31 | 100% (5/5) |
| *K. pneumoniae* (hypermucoviscous) *mag*A |  | 28.23 | 100% (5/5) |
| *C. auris* |  | 31.05 | 100% (5/5) |
| CI |  | 28.40 | 100% (5/5) |
